# Characterization of the Olfactory Receptor OR10H1 in Human Urinary Bladder Cancer

**DOI:** 10.3389/fphys.2018.00456

**Published:** 2018-05-16

**Authors:** Lea Weber, Wolfgang A. Schulz, Stathis Philippou, Josephine Eckardt, Burkhard Ubrig, Michéle J. Hoffmann, Andrea Tannapfel, Benjamin Kalbe, Günter Gisselmann, Hanns Hatt

**Affiliations:** ^1^Department of Cellular Physiology, Ruhr University Bochum, Bochum, Germany; ^2^Department of Translational Wound Research, Witten/Herdecke University, Witten, Germany; ^3^Department of Urology, Medical Faculty, Heinrich Heine University Düsseldorf, Düsseldorf, Germany; ^4^Department of Pathology and Cytology, Augusta Kliniken Bochum Hattingen, Bochum, Germany; ^5^Institute for Physiology, Ruhr University Bochum, Bochum, Germany; ^6^Clinic for Urology, Augusta Kliniken Bochum Hattingen, Bochum, Germany; ^7^Institute for Pathology, Ruhr University Bochum, Bochum, Germany

**Keywords:** olfactory receptor, bladder, bladder cancer, biomarker, next generation sequencing, OR10H1

## Abstract

Olfactory receptors (ORs) are a large group of G-protein coupled receptors predominantly found in the olfactory epithelium. Many ORs are, however, ectopically expressed in other tissues and involved in several diseases including cancer. In this study, we describe that one OR, OR10H1, is predominantly expressed in the human urinary bladder with a notably higher expression at mRNA and protein level in bladder cancer tissues. Interestingly, also significantly higher amounts of OR10H1 transcripts were detectable in the urine of bladder cancer patients than in the urine of control persons. We identified the sandalwood-related compound Sandranol as a specific agonist of OR10H1. This deorphanization allowed the functional characterization of OR10H1 in BFTC905 bladder cancer cells. The effect of receptor activation was morphologically apparent in cell rounding, accompanied by changes in the cytoskeleton detected by β-actin, T-cadherin and β-Catenin staining. In addition, Sandranol treatment significantly diminished cell viability, cell proliferation and migration and induced a limited degree of apoptosis. Cell cycle analysis revealed an increased G1 fraction. In a concentration-dependent manner, Sandranol application elevated cAMP levels, which was reduced by inhibition of adenylyl cyclase, and elicited intracellular Ca^2+^ concentration increase. Furthermore, activation of OR10H1 enhanced secretion of ATP and serotonin. Our results suggest OR10H1 as a potential biomarker and therapeutic target for bladder cancer.

## Introduction

Bladder cancer is the second most common genitourinary cancer type in the world, with at least 429,000 newly diagnoses made per year. Approximately 70–80% of the patients have non-muscle invasive urothelial carcinoma, but 20–30% of the tumors are invasive urothelial carcinomas or – more rarely – other aggressive subtypes (Ferlay et al., 2015; Torre et al., 2015). Although several studies analyzing the molecular profile of invasive urothelial carcinomas have revealed potential targets for drug therapies, no molecularly targeted agent is currently used for the treatment of this malignancy (The Cancer Genome Atlas Research Network, 2014). Recently, the use of immune checkpoint inhibitors has been shown to improve the treatment of metastatic urothelial cancers, but overall treatment options remain limited (Kamat et al., 2016). Therapy for invasive urothelial cancer frequently includes radical surgical removal of the bladder as well as cisplatin-based chemotherapy, but the therapeutic efficacy of the latter is limited by drug resistance mechanisms. Hence, new therapeutic approaches are urgently needed. Here, we investigated the potential of olfactory receptors (ORs) in this respect. ORs belong to the class of G protein-coupled receptors (GPCRs), representing the largest supergene family in the human genome. The canonical olfactory signaling pathway involves adenylyl cyclase, which leads to an increase of intracellular cAMP and to a subsequent influx of calcium into the cell (Bakalyar and Reed, 1991). Typically, ORs detect volatile odorant molecules in the olfactory epithelium of the nose (Buck and Axel, 1991). Recent studies have provided evidence for a physiological role of ORs outside the olfactory epithelium as well as a crucial influence on several diseases, including cancer (Veitinger and Hatt, 2017). ORs can be detected in a broad range of healthy human tissues (Kang and Koo, 2012; Flegel et al., 2013) as well as cancer tissues. This was first observed in prostate cancer, where activation of OR51E2 by its ligand β-Ionon leads to diminished cell proliferation and migration (Neuhaus et al., 2009). Similar effects could be demonstrated in melanoma cells (Gelis et al., 2017). In the lung, OR2J3 is capable of reducing cell proliferation upon stimulation with Helional (Kalbe et al., 2017). Similarly, OR2AT4 induced apoptosis in leukemia when stimulated with Sandalore (Manteniotis et al., 2016). In hepatocellular carcinoma cells, the receptor OR1A2 reduced cell proliferation when activated by the monoterpene (-)-Citronellol (Massberg et al., 2015). In colorectal cancer, OR51B4 is capable of reducing cell proliferation and migration and inducing apoptosis upon application of its ligand Troenan (Weber et al., 2017). Thus, ORs are capable of modulating several important cellular processes in cancer cells, and might be considered as novel targets for therapy. Due to their specific expression patterns, some ORs could moreover serve as tumor biomarkers, such as OR51E1 and OR51E2, in prostate cancer (Xu et al., 2000; Neuhaus et al., 2009; Massberg et al., 2016), lung cancer (Giandomenico et al., 2013) and small intestine cancer (Cui et al., 2013) and OR7C1 as a marker of cancer-initiating cells in colorectal cancer (Morita et al., 2016). The lack of activating ligands for most ORs is still the bottleneck for further studies on their physiological and pathophysiological roles in different human tissues. Several studies have revealed tissue-specific expression profiles for ORs but the functions in the expressing tissues remain unknown (Flegel et al., 2013; Morita et al., 2016; Veitinger and Hatt, 2017). Therefore, deorphanization of ORs is of major importance.

In the present study, we describe expression of the OR OR10H1 in bladder cancer. OR10H1 is predominantly expressed in the urinary bladder, among all tissues, with increased expression in many bladder cancer tissues. High expression rates of the receptor were shown for several bladder cancer cell lines and many native cancer tissues, but also increased during differentiation of urothelial cells in culture. Transcripts of OR10H1 are detectable at significantly higher levels in the urine of bladder cancer patients. We also report the identification of the odorant Sandranol, which renders a typical sandal note, as an agonist of OR10H1, which stimulates canonical responses like calcium influx and cAMP increase, mediated by adenylyl cyclase 3. In a bladder cancer cell line, we demonstrate that Sandranol stimulation significantly inhibits cell proliferation, migration, induces cell cycle arrest in G1 and -albeit to a limited extent- apoptosis.

## Materials and Methods

### Chemicals

The odorants for the deorphanization studies were a kind gift from Symrise AG (Holzminden, Germany) and Henkel (Düsseldorf, Germany). Forskolin and SQ22536 were purchased from Sigma Aldrich (Munich, Germany). The odorants and inhibitors were prediluted or dissolved in DMSO prior to usage.

### Plasmids

For the transfection of Hana3A cells, we constructed an expression plasmid containing OR10H1. This plasmid expressed OR10H1 as a fusion protein with an N-terminal rhodopsin tag. Standard PCR methods were used to construct the rho-tagged pCI expression vectors coding for OR10H1 as previously described (Saito et al., 2004).

### Next Generation Sequencing

We used the TruSeq^TM^ RNA Sample Prep Kit v2 according to the manufacturer’s protocol for standard mRNAseq (Illumina, San Diego, CA, United States). RNA-Seq was conducted on the HiSeq2000 (2 × 102 bp reads) sequencing platform. The parameters for the analysis were the same as described in Weber et al. (2017). Version hg19 of the human genome and transcriptome was used for the read alignment in TopHat (v.1.2.0). The arrangement of the alignment was performed by the short-read mapping program Bowtie that is included in TopHat. The resulting output files, in BAM format, were sorted and indexed by the program SAMtools, whereas the calculation of the abundance of the aligned mRNA-Seq reads was conducted by the program Cufflinks. Cufflinks uses the RefSeq hg19 reference transcriptome in Gene Transfer Format (GTF) received from the UCSC Genome Bioinformatics database (University of California, Santa Cruz).

### Raw RNA-Seq Data

Data sets used for RNA-Seq analysis were taken from the SRA-archive, NCBI. For further details see **Supplementary Table S2**.

### RNA Isolation From Cell Lines

Cells were seeded and incubated for 24 h. Then, cells were harvested and the cell pellet was disrupted using a homogenizer (Percellys24) and 1.4. ceramics beads (Precellys^®^). The RNeasy Mini Kit (Qiagen, Hilden, Germany) was used to isolate RNA according to the manufacturer’s protocol, including G-Eliminator columns and an additional on-column DNaseI digestion.

### RNA Isolation From Urine

Human urine samples were obtained from Dr. Ubrig and Dr. Scheer at the Augusta-Kranken-Anstalt Bochum. The study was carried out in accordance with the Declaration of Helsinki. Each patient, who participated, signed a written informed consent. The experiments were approved by the Regional Research Ethics Committee (Ethical Committee of the Medical Faculty, Ruhr-University Bochum); Ethic no: 17-6116. RNA was isolated from urinary cells as described by Mengual and Olivan (2018).

### Reverse Transcriptase PCR (RT-PCR)

The expression of OR10H1 was validated by RT-PCR, using primers spanning both exons of OR10H1 to avoid artifacts from DNA-contamination. The PCR product encompassed 269 bp. RT-PCR was conducted using the GoTaq qPCR Master Mix (Promega, Madison, WI, United States) or the Qiagen Quantitec Master Mix (Qiagen, Hilden, Germany), according to the manufacturer’s instructions. *TBP* was used as a reference gene. Standard curves were run for each gene to ensure appropriate PCR efficiency and relative expression was then calculated by the Δ*C*q method. For details on all primers see **Supplementary Table S1**.

### Cell Culture

A series of bladder cancer cell lines, representing different stages and grades of the disease, were used as described previously (Koutsogiannouli et al., 2017). Most of the functional experiments were conducted using the moderately differentiated cell line BFTC905, which was established from a grade 2 stage C urothelial carcinoma (Cheng et al., 1995). UCC cell lines were cultured in DMEM GlutaMAX-I (Gibco^®^, Life Technologies, Darmstadt, Germany) supplemented with 10% fetal bovine serum (FBS) (GE Healthcare, Piscataway, NJ, United States). HBLAK cells were cultivated in CnT-Prime Epithelial Culture Medium (CELLnTEC, Bern, Switzerland) as previously described (Hoffmann et al., 2016). hTERT-NHUC cells, generously provided by Dr. M. A. Knowles, Leeds, were cultivated in keratinocyte serum-free medium (Gibco^®^, Life Technologies, Darmstadt, Germany) supplemented with 0.25 ng/mL EGF, 12.5 μg/mL bovine pituitary extract, 0.35 μg/mL of N-epinephrine, 0.33 mg/mL hydrocortisone (Sigma-Aldrich, Munich, Germany) and 1:100 insulin-transferrin-selenium (Gibco^®^, Life Technologies). Hana3A cells were a kind gift of Prof. Dr. H. Matsunami. The cultivation medium for BFTC905 and Hana3A cells was Dulbecco’s Modified Eagle’s medium (DMEM) supplemented with 10% FBS, 2 mM glutamine and 100 units/ml penicillin/streptomycin (Gibco^®^, Life Technologies, Carlsbad, CA, United States). The cells were cultured in a humidified atmosphere (37°C, 6% CO_2_) and were passaged every 3 days at a confluence of 90%. TrypLE^TM^ (Life Technologies, Carlsbad, CA, United States) was used for cell detachment. Hana3A cells were used for the deorphanization studies in the CRE-Luciferase assay, because they stably express REEP1, RTP1L, RTP2, and Gα_ol,_ supporting the stable heterologous expression of ORs (Saito et al., 2004).

### Immunocytochemistry (ICC) and Immunohistochemistry (IHC)

For the immunocytochemical staining the following antibodies were used: rabbit polyclonal anti-OR10H1 antibody (OriGene, Herford, Germany; dilution: 1:100), rabbit polyclonal anti-CadherinT antibody (Cell Signaling, Leiden, Netherlands; dilution: 1:50), mouse monoclonal α-E-cadherin (Cell Signaling, Cambridge, United Kingdom, 1:100), mouse monoclonal α-Rhodopsin 4D2 antibody (AR441, Dako, Glostrup, Denmark; dilution: 1:100). For staining of β-actin Phalloidin was used (Santa Cruz, Heidelberg, Germany; dilution: 1:100). The immuncytochemical staining of the transfected Hana3A and BFTC905 cells was performed as previously described (Weber et al., 2017).

The immunohistochemical staining of human bladder cancer and normal bladder tissues was conducted using a Ventana BenchMark Ultra instrument (Ventana Medical System, Tucson, AZ, United States) and the UltraView Universal DAB detection Kit according to the manufacturer’s instructions. The primary antibody OR10H1 was diluted in antibody diluent (1:20) and applied for 32 min. All sections were counter-stained with haematoxylin (HE). An Olympus BX 43 microscope was used for visualization.

### pmir GLO Dual-Luciferase Assay

For deorphanization of OR10H1 we used the Dual-Luciferase assay optimized for OR screening by Zhuang and Matsunami (2008). For further details see (Weber et al., 2017). We used Henkel 100 for an efficient screening procedure. Henkel 100 contains aliphatics, alcohols, aromatics, amines, alkanes, aldehydes, esters, ethers, ketones, heterocyclics and others, and thus, ensures the activation of as many ORs as possible by the broad range of different chemical compounds. For further details and the odorant list see Hatt et al. (1999).

### Calcium Imaging

The BFTC905 cells were seeded overnight in 35-mm dishes and were incubated at 37°C for 30 min with 3 μM FURA-2-AM (fura-2-acetoxymethyl ester, Molecular Probes, Eugene, OR, United States). After the incubation, the growth medium was replaced with Ringer’s solution (containing 140 mM NaCl, 2 mM CaCl_2_, 5 mM KCl, 1 mM MgCl_2_ and 10 mM HEPES at pH 7.4) and fluorimetric imaging was performed as previously described (Soloway et al., 1996). The BFTC905 cells were exposed to 100, 300, and 500 μM Sandranol (2–3 times for 30–60 s, depending on the experimental approach). For this purpose, 5 M Sandranol stock was prediluted in DMSO and dissolved in Ringer’s solution to its final concentrations. The final concentration of the DMSO solvent was <0.1%.

### Serotonin Assay

BFTC905 cells were seeded in 96-well plates and were incubated overnight. The cells were washed twice with PBS and incubated in Ringer’s solution, containing Fluoxetine (2 μM), Sandranol (300, 500, and 700 μM) or control (DMSO) for 24 h. The resulting serotonin levels were analyzed using an enzyme immunoassay kit according to the manufacturer’s protocol (5-HT ELISA, Enzo Life Sciences AH Diagnostic AB, Solna, Sweden).

### cAMP Assay

The intracellular cAMP level of BFTC905 cells was monitored via the cAMP-Glo^TM^ assay by Promega (Madison, WI, United States). BFTC905 were seeded on a 96 well plate (Thermo Fisher Scientific, Waltham, MA, United States) at a density of 20,000 cells per well 1 day prior to the experiment. Cells were washed in induction buffer (Ringer’s solution containing 500 μM IBMX and 100 μM Ro 20-1724) and incubated with Sandranol alone or Sandranol with the specific adenylyl cyclase inhibitor SQ22536 (100 μM, Abcam, Cambridge, United Kingdom) diluted in induction buffer for 20 min. As controls, 0.1% DMSO or 0.1% DMSO with 100 μM SQ22536 were used. The assay was conducted according to the manufacturer’s protocol. Luminescence was monitored via a plate reader (Packard, PerkinElmer, Waltham, MA, United States) and relative light units were normalized to the DMSO (0.1%) control.

### ATP-Based Cell Viability and Apoptosis Assay

Total cellular ATP, as an indicator for cell viability, was measured using the CellTiter-Glo Luminescent Cell Viability Assay (Promega, Mannheim, Germany). 7.5 × 10^4^ BFTC905 cells per well were seeded in 6-well plates, cultured overnight, treated with Sandranol (50, 100, 300, 500, and 700 μM) and grown for 24 or 48 h. Then, cell aliquots were transferred into 96-well plates and viability was measured using the CellTiter-Glo Reagent. Apoptosis was quantified by the Caspase-Glo 3/7 assay (Promega, Mannheim, Germany) and was normalized to the ATP assay data.

### Flow Cytometry

To follow changes in cell cycle and apoptosis via flow cytometry, 7.5 × 10^4^ BFTC905 cells were seeded in 6-well culture plates and were incubated for 24 h before incubation with Sandranol for another 24 or 48 h. The attached and floating cells were stained using PI-buffer containing 50 μg/ml propidium iodide, 0.1% Triton X-100 and 0.1% sodium citrate and flow cytometry was performed using a Miltenyi MACSQuant Analyzer (Miltenyi Biotec GmbH, Bergisch Gladbach, Germany). The resulting DNA histograms were fitted according to a standard method (Ormerod, 2001), using the MACSQuantify software (Miltenyi Biotec GmbH, Bergisch Gladbach, Germany).

### Culture and Induced Differentiation of Normal Urothelial Cells

Primary cultures of normal urothelial cells were established from healthy ureters removed during tumor nephrectomy and were cultured as previously described (Swiatkowski et al., 2003) in KSFM medium supplemented with 5 ng/ml of EGF and 50 μg/ml of bovine pituitary extract (Life Technologies, Carlsbad, CA, United States). Differentiation was induced following published protocols (Varley et al., 2004; Cross et al., 2005) and was ascertained by morphological changes and specific mRNA marker expression as previously described (Hoffmann et al., 2016).

### Scratch Assay

Cell migration in the BFTC905 cells was analyzed using an *in vitro* wound scratch assay. Here, the BFTC905 cells were seeded and incubated for 24 h. Confluent monolayers of BFTC905 cells were scratched using a 10-μl pipette tip, washed with PBS and incubated with DMEM containing Sandranol (10, 50, and 100 μM). The size of the residual gap was measured after 24 and 48 h and the software *tscratch* was used to calculate the overgrown cell area relative to the initial scratch area (Geback et al., 2009).

### Cell Proliferation – EdU

Cell proliferation was quantified using the EdU HTS Kit (Sigma-Aldrich, St. Louis, MO, United States) according to the manufacturer’s protocol.

### Statistics

All the results were tested for normality (Shapiro–Wilk) and equal variance. For the data passing the tests, we used a two-tailed unpaired *t*-test. The data that were not normally distributed were analyzed using the Mann–Whitney Rank Sum Test. The results of the cAMP assays were statistically analyzed using a One-Way Repeated Measures ANOVA with a *posthoc* Tukey test. Unless stated otherwise, the values represent the mean ± SEM (standard error of the mean) from at least three independent experiments. Statistical significance was indicated as follows: ^∗^*p* < 0.05, ^∗∗^*p* < 0.01, ^∗∗∗^*p* < 0.001.

## Results

### OR Expression Profile in Cancer Tissues

**FIGURE 1:**
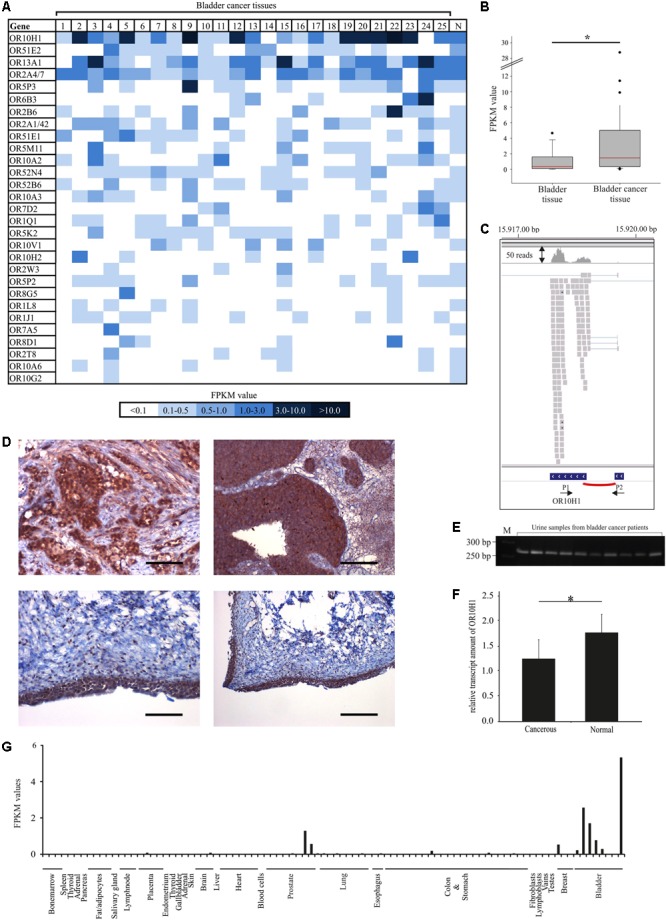
Expression pattern of OR10H1 and other ectopically expressed ORs in bladder cancer tissues. **(A)** The heat map shows the FPKM values of the 30 most highly expressed ORs found in 25 different human bladder cancer tissues and in the healthy bladder (*N*). Healthy bladder = average expression in five different healthy bladder tissues. FPKM values are visualized by color depth; dark blue indicates FPKM values > 3 and light blue indicates FPKM values < 0.5. **(B)** Box-plot showing the expression of OR10H1 in normal bladder tissues (*n* = 11) and bladder cancer tissues and – cell lines (*n* = 25). **(C)** Read coverage of OR10H1 detected in bladder cancer tissues and visualized by the Integrative Genomic Viewer. Reads are visualized as gray squares. Splicing is shown as red arc. Bottom: Arrows show the localization of the intron-spanning PCR Primers (P1, P2). **(D)** Protein expression of OR10H1 in human bladder cancer tissues. Top left: IHC of an urothelial carcinoma tissue with glandular differentiation. The expression of OR10H1 is localized only in cancerous cells. Scale bar: 200 μm, enlarged: 200×. Top right: IHC of an urothelial bladder carcinoma tissue. Scale bar: 100 μm, enlarged: 100×. Bottom left and right: Normal bladder urothelial tissue. Left: scale bar: 100 μm, enlarged: 100×, right: scale bar: 20 μm, enlarged: 20×. DAB chromogenic staining was used for the visualization of protein expression. HE was used to reveal the tissue architecture. **(E)** Detection of OR10H1 transcript in 10 human urine samples from patients with bladder cancer, determined by RT-PCR. M, marker. **(F)** Bar chart showing the amount of OR10H1 transcript relative to TATA Box binding protein (TBP) as a reference gene (see section “Materials and Methods” for details) in human urine samples from patients suffering from bladder cancer (*n* = 19) and from healthy persons (*n* = 25). The relative transcript amount was calculated using TBP as reference gene. ^∗^*p* < 0.05. **(G)** Expression of OR10H1 in different normal tissues (other tissues: *n* = 83, bladder tissues/cell lines: *n* = 8). For detailed information on datasets see **Supplementary Table S2**.

To profile the expression of OR in bladder cancer tissues, we investigated RNA-Seq data from 25 bladder cancer tissues as well as corresponding normal tissues for the expression of OR genes. To this end, we reanalyzed existing RNA-Seq data from the NCBI archive with a focus on the expression of ORs (**Figure 1A**).

OR10H1 was expressed in 23 out of 25 bladder cancer tissue samples, with an average FPKM-value of 3.8, whereas it was only moderately expressed in the normal bladder (*n* = 5) with an average value of 0.3 (**Figure 1A**). None of the other 28 ranked ORs showed a similarly high expression in bladder cancer. The specificity of OR10H1 expression for bladder tissue is underlined by analysis of the GTex data [The Genotype-Tissue Expression (GTEx) project 2013], according to which OR10H1 is prominently expressed in the bladder, but is barely expressed in other tissues. The statistical analyses, including 11 data sets of healthy bladder tissues from the GTex database revealed that the expression between healthy and cancerous tissue differed significantly (**Figure 1B**). In line with these results, data from the BioXpress database (Wan et al., 2015) revealed that OR10H1 also showed the highest expression in bladder cancer tissues compared to 26 other cancer tissues (data not shown). An analysis of the read coverage of OR10H1 by the Integrative Genomic Viewer validated the calculation of the FPKM values by means of the read distribution on the two exons of OR10H1 (**Figure 1C**).

Validation of OR10H1 protein expression was performed via immunohistochemical staining with a specific OR10H1-detecting antibody in bladder cancer tissues (**Figure 1D**). Its specificity was validated in OR10H1-transfected Hana3A cells (**Supplementary Figure S1**). The staining revealed a strong expression in the carcinoma cells of cancerous tissues, but not in stromal cells, and weaker expression in normal urothelium (**Figure 1D**). Thus, OR10H1 showed a specific expression in bladder urothelium with a higher expression in many bladder cancer tissues than in normal tissue.

Next, we investigated whether OR10H1 mRNA can be detected in urine from healthy human donors and patients with bladder cancer. Urine samples from patients with bladder cancer revealed a significantly higher amount of OR10H1 transcripts than urine samples from healthy donors (**Figures 1E,F**).

To verify the specificity of OR10H1 expression for bladder, we analyzed transcriptome data across several human tissues from the SRA-archive. Again, the expression of OR10H1 was minimal in nearly all tissues except bladder and prostate, which contains a urethra segment with urothelial lining (**Figure 1G**).

### OR10H1 Expression in Human Bladder Cancer Cell Lines

**FIGURE 2:**
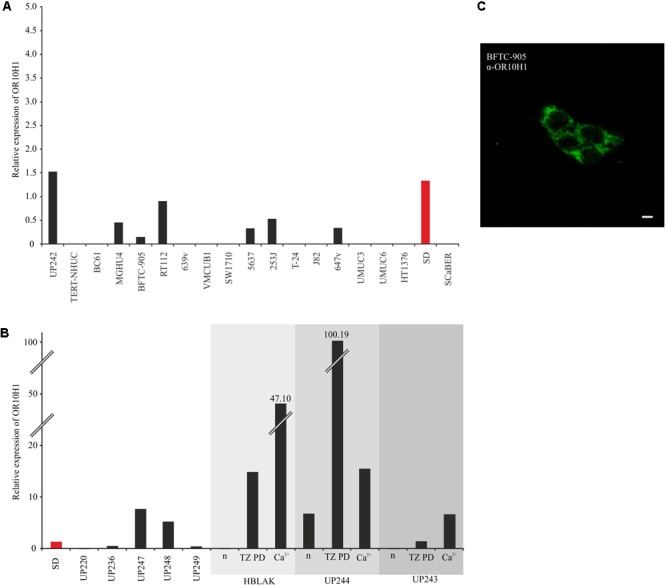
Expression of OR10H1 in cell lines originated from normal bladder tissues (UP) and cancerous bladder tissues. **(A)** mRNA expression of OR10H1 in different urothelial carcinoma cells and normal urothelial cells. Carcinoma cell lines: BC61, MGHU4, BFTC-905, RT112, 639v, VMCUB1, SW1710, 5637, 253J, T-24, J82, 647v, UMUC3, UMUC6, HT1376, SD. Normal urothelial cells: TERT-NHUC. **(B)** Expression of OR10H1 in independent primary cultures of normal urothelial cells (UP) and following induction of differentiation in UPs or the immortalized benign urothelial cell line HBLAK by two different protocols (TZ/PD or Ca^2+^). OR10H1 expression was determined by qRT-PCR and was adjusted to TBP mRNA. Expression in SD (red) was measured in **(A,B)** for comparison. **(C)** Expression of OR10H1 in BFTC905 cells. Immunocytochemical staining of OR10H1 in BFTC905 cells with a specific OR10H1-antibody. Scale bar: 10 μm.

To find a suitable model for analyzing the function of OR10H1, we investigated the expression of OR10H1 by qRT-PCR in a large panel of bladder cancer cell lines representing different stages and grades of the disease, and in normal control cells, namely, primary cultures of proliferating normal uroepithelial cells (UPs), and two immortalized cell lines derived from these, HBLAK (Hoffmann et al., 2016) and TERT-NHUC (Chapman et al., 2009). OR10H1 expression was detectable in several UC cell lines, especially, but not exclusively in better differentiated, epitheloid cell lines, such as BFTC-905, RT112 and 5637 (**Figure 2A**). In many others, OR10H1 expression was undetectable, especially in poorly differentiated cell lines with a mesenchymal phenotype. Among individual UP cultures, expression was variable, but was undetectable in HBLAK and TERT-NHUC cells under basal growth conditions (**Figure 2B**).

Since UP cultures consist largely of proliferating precursor cells with a basal phenotype, but contain varying admixtures of more differentiated cells, we investigated the expression of OR10H1 during induced differentiation of UPs and HBLAK cells. OR10H1 expression was strongly induced by all differentiation protocols employed. For further experiments, we used the cell line BFTC905 and validated OR10H1 expression by immunocytochemistry using a specific OR10H1-antibody (**Figure 2C**).

### Deorphanization of OR10H1

**FIGURE 3:**
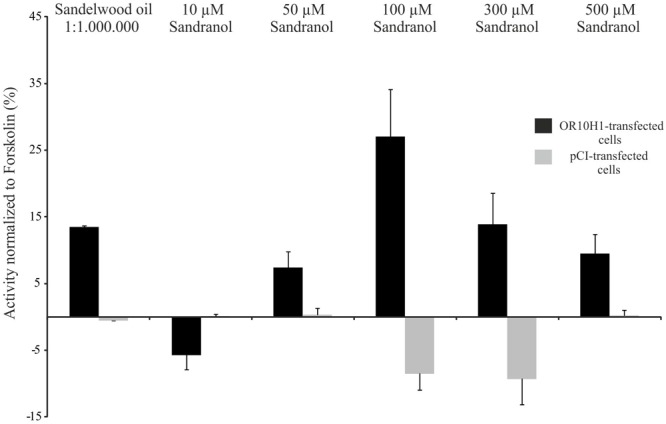
Luciferase activity in OR10H1-transfected Hana3A-cells. Activation of OR10H1-transfected Hana3A cells by Sandranol. Bar chart showing normalized luminescence values upon activation with Sandranol. OR-transfected cells: Hana3A cells transfected with pCI-vector containing OR10H1. pCI-transfected control cells: Hana3A cells transfected with pCI-vector alone.

Since OR10H1 is an orphan receptor, we conducted deorphanization experiments using the established CRE-Luciferase reporter gene assay in Hana3A cells which express several accessory proteins necessary for the membrane targeting of ORs (Saito et al., 2004). The adequate expression of OR10H1 in Hana3A cells transfected with the OR10H1 expression plasmid was verified by immunocytochemical staining (**Supplementary Figure S1**). Applying the Henkel100 mixture, which contains 100 structurally different odorous substances, we detected a slight activation of OR10H1-transfected Hana3A cells. By subdividing the mixture we identified the synthetic sandalwood odorant Sandalore as an agonist effective only in high concentrations (data not shown). Accordingly, natural sandalwood oil elicited a cAMP increase as well (**Figure 3**). By testing further synthetic sandalwood odorants we detected that Sandranol significant increased the cAMP level and induced cAMP-dependent luciferase expression (**Figure 3**). Other substances did not evoke an increase in cAMP.

Since Sandranol also elicited a significant luciferase activity at concentrations > 50 μM, it was used as ligand for OR10H1 in further experiments.

### Physiological Effects of Sandranol Stimulation on BFTC905 Cells

**FIGURE 4:**
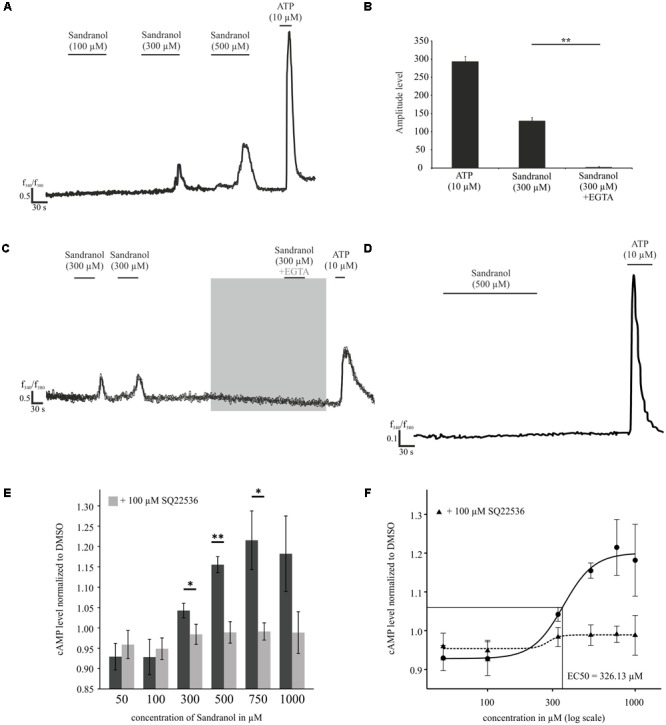
Physiological effects of Sandranol application on BFTC905 cells. **(A)** Representative responses of BFTC905 cells stimulated with Sandranol in calcium imaging experiments. Application of Sandranol at concentrations of 100, 300, and 500 μM induced a dose-dependent calcium increase. **(B)** Bar chart showing mean amplitudes of Sandranol-induced Ca^2+^ signals in BFTC905 cells. **(C)** Localization of Ca^2+^ in BFTC905 cells upon addition of ATP, Sandranol or Sandranol plus EGTA in Ringer’s solution. *N* = 64. **(D)** Representative trace of untreated HANA3A cells stimulated with Sandranol (500 μM). **(E)** Concentration-dependent increase of the cAMP level upon stimulation with Sandranol (50, 100, 300, 500, 750, and 1000 μM) (*N* = 3 – 6). Dark gray bars represent the increase of the cAMP level induced by Sandranol alone on BFTC905 cells and light gray bars demonstrate the effect after co-incubation with the adenylyl cyclase inhibitor SQ22536 (100 μM). Significance was tested using One-Way repeated measures ANOVA with a Tukey’s *post hoc* test. **(F)** Data from **(E)** were plotted in a concentration response curve; using the Hill equation yielded an EC_50_ of 326 μM. ^∗^*p* < 0.05, ^∗∗^*p* < 0.01.

Next, we investigated the effect of Sandranol stimulation on OR10H1 expressing BFTC905 cells. First, we investigated whether application of Sandranol influences Ca^2+^ homeostasis in BFTC905 cells. Calcium imaging analysis revealed that Sandranol, at different concentrations, induced dose-dependently increasing amplitudes of calcium influx into the cells (**Figure 4A**). Up to 20% of all BFTC905 cells showed increased calcium levels by Sandranol at 300 μM. Addition of EGTA to the incubation medium diminished the transient increase in the intracellular calcium concentration upon Sandranol stimulation indicating its dependence on extracellular calcium (**Figures 4B,C**). As a control experiment, we stimulated untreated HANA3A cells with Sandranol and could not observe any effects on the intracellular calcium level (**Figure 4D**). Furthermore, the intracellular cAMP level of BFTC905 cells was increased by Sandranol in a dose-dependent fashion with an EC_50_ of 326 μM (**Figure 4F**). The Sandranol-induced cAMP elevation was strongly diminished by the adenylyl cyclase (AC)-specific inhibitor SQ22536 (100 μM) (**Figures 4E,F**).

**FIGURE 5:**
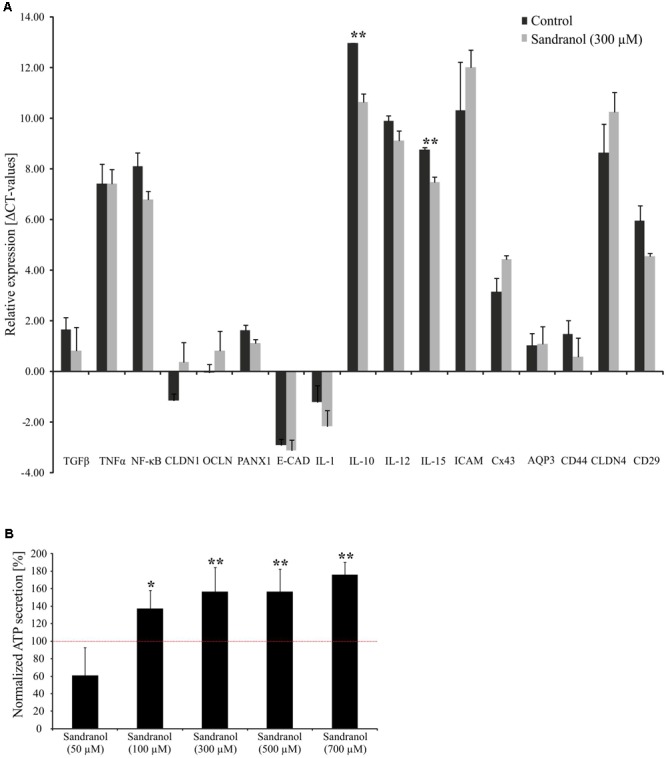
Physiological effects on BFTC905 cells upon stimulation with Sandranol. **(A)** Delta CT-values of RT-PCR assays for various interleukins and cell adhesion molecules in BFTC905 cells stimulated with Sandranol (300 μM) for 24 h. CLDN1, Claudin-1; OCLN, Occludin; PANX1, Pannexin-1; E-CAD, E-Cadherin; IL-1, Interleukin-1; IL-10, Interleukin-10; IL-12, Interleukin-12; IL-15, Interleukin-15; ICAM, ICAM-1, Intercellular Adhesion Molecule 1; Cx43, Connexin 43; AQP3, AquaPorin-3; CLDN4, Claudin-4; CD29, Integrin B1. Amount of OR10H1 transcript is calculated relative to TATA Box binding protein (TBP) as a reference gene. **(B)** Induction of a dose-dependent increase in ATP in BFTC905 cells upon Sandranol stimulation after 24 h. ^∗^*p* < 0.05, ^∗∗^*p* < 0.01.

We analyzed the physiological effects of Sandranol on cellular mediators in BFTC905 cells by different assays. First, we investigated the effect of Sandranol stimulation on the expression of various cytokines and cell adhesion molecules via qRT-PCR. Among the cytokines, the transcript levels for IL-10 and IL-15 increased significantly after incubating the BFTC905 cells with Sandranol (300 μM), whereas the expression of other cytokines, including TGFβ, TNFα, IL-1, IL-12, and NF-κB did not change (**Figure 5A**). Likewise, the transcript levels of cell adhesion and transmembrane proteins Claudin-1, Occludin, Pannexin-1, CAD, Intracellular Adhesion Molecule 1, Connexin 43, CD44, Claudin 4 and AquaPorin-3 were unaffected.

We further analyzed the amounts of serotonin and ATP, which are common signaling molecules in the bladder (Burnstock, 2014), by a colorimetric ATP Assay Kit and a serotonin ELISA, respectively. ATP secretion increased with increasing odorant concentration (**Figure 5B**). Likewise, stimulating BFTC905 cells with Sandranol for 24 h increased the serotonin concentration in the cell medium (**Supplementary Figure S2**).

**FIGURE 6:**
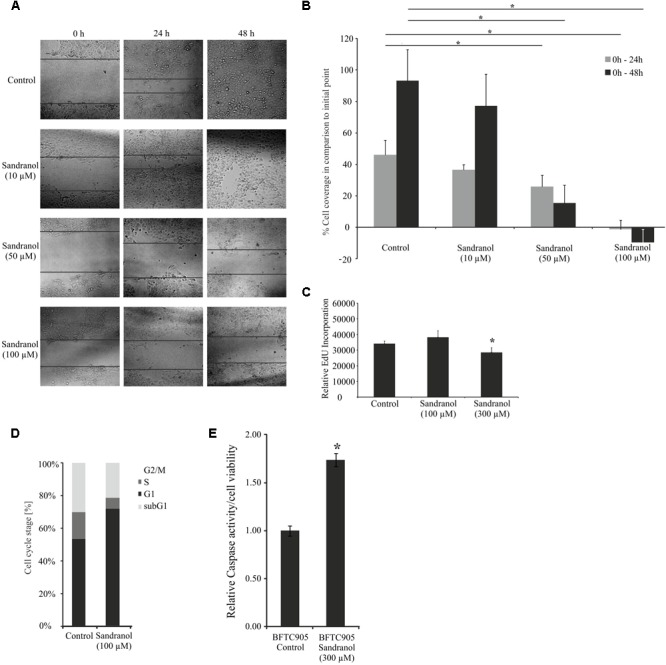
Impact of Sandranol on cellular properties of BFTC905 cells. **(A)** Analyses of cell migration by a scratch assay after Sandranol stimulation (10, 50, and 100 μM) for 24 and 48 h. Bar chart shows the statistical analysis of the area overgrown in scratch assay experiments. *N* = 3 assays. **(B)** Bar chart showing statistical analysis of area overgrown by BFTC905 cells in scratch assay experiments. Gray: treatment for 24 h, black: treatment for 48 h. *N* = 3 assays. **(C)** Proliferation analyses by EdU incorporation of BFTC905 cells after stimulation with Sandranol (100 and 300 μM). **(D)** Cell cycle analysis of BFTC905 cells after stimulation with Sandranol (100 μM). All cell cycle phases were significantly different in treated cells. **(E)** Cell viability in BFTC905 cells induced by Sandranol (300 μM) treatment for 24 h. Bar chart showing relative caspase activity in the BFTC905 cells. ^∗^*p* < 0.05.

After having demonstrated specific physiological effects of Sandranol on BFTC905 cells, we investigated whether the compound impacted cellular properties that were relevant for cancer progression, such as migration, proliferation and cell cycle distribution. For the investigation of cell migration, Sandranol (10, 50, and 100 μM) was applied to BFTC905 cells, and cell migration was measured after 24 and 48 h via a scratch assay. Incubation with Sandranol at active concentrations (50 and 100 μM) led to a significant reduction in cell migration after both time periods (**Figures 6A,B**). The effect of Sandranol on cell proliferation was analyzed by an EdU incorporation assay. Incorporation of EdU, as an indicator of DNA synthesis, was diminished 24 h after starting the treatment with Sandranol (**Figure 6C**). The same effect was observed for Santalol, the main component of natural sandalwood oil (**Supplementary Figure S3**). Accordingly, as measured by flow cytometry, Sandranol induced a pronounced increase in the G1 fraction and accordingly decreased the S-phase and G2/M-fractions in the cell line BFTC-905. A slight increase in the very small sub-G1 fraction was additionally observed (**Figure 6D**). In keeping with this observation, caspase 3/7 activity was slightly, but was significantly enhanced by 75% in Sandranol-treated BFTC905 cells (**Figure 6E**).

**FIGURE 7:**
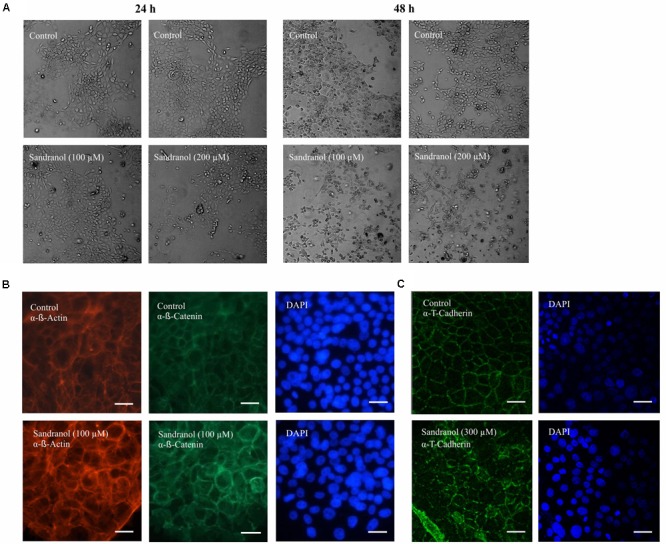
BFTC905 cell morphology after Sandranol treatment. **(A)** Morphological changes in BFTC905 cells induced by Sandranol (100 μM/200 μM) after 24 (left) and 48 h (right). **(B)** Immunocytochemical staining of the Sandranol-treated BFTC905 cells (100 μM for 24 h) with antibodies detecting β-Catenin and phalloidin (α-β-Actin) staining for detecting cytoskeletal β-Actin. **(C)** Immunocytochemical staining of Sandranol-treated BFTC905 cells (300 μM for 24 h) with an antibody detecting T-Cadherin (green, dilution: 1:100). DAPI was used to visualize the nuclei of BFTC905 cells. Scale bar: 50 μm.

Stimulating BFTC905 cells with Sandranol (300 μM) for 24 and 48 h led to changes in cell morphology (**Figure 7A**). Immunocytochemical staining of BFTC905 cells with antibodies detecting T-Cadherin and β-Catenin showed a reorganization of the cell cytoskeleton leading to cell rounding (**Figures 7B,C**). Compared to control cells, Sandranol stimulation disrupted the regular uniform cytoskeleton structure and often diminished cell-to-cell adhesion.

## Discussion

Since ORs were discovered outside the olfactory epithelium, knowledge on the physiological and pathophysiological effects of ectopically expressed ORs has been accumulating continuously. Ectopically expressed ORs become more relevant as several physiological functions have been determined. They influence sperm motility and chemotaxis (Spehr et al., 2003), act on the serotonin release of enterochromaffin cells in the gut (Braun et al., 2007), mediate the regeneration of keratinocytes after wounding (Busse et al., 2014), or induce negative chronotropic effects in cardiomyocytes (Jovancevic et al., 2017).

Furthermore, the influence of ORs on cancer progression and their potential for diagnosis has been demonstrated for many different cancers (Neuhaus et al., 2009; Cui et al., 2013; Giandomenico et al., 2013; Massberg et al., 2015, 2016; Manteniotis et al., 2016; Rodriguez et al., 2016; Gelis et al., 2017; Kalbe et al., 2017; Weber et al., 2017). In the present study, the detection of OR10H1 mRNA and protein via NGS, RT-PCR and immunocytochemistry in bladder tissues revealed a significantly higher expression in cancerous tissues compared to normal bladder. Strikingly, OR10H1 was highly specific for bladder tissues, as this OR was virtually absent in other normal and cancerous tissues. This suggests that OR10H1 might be a potential biomarker that is specific for bladder cancer. Further studies should address the relationship between OR10H1 expression and the recently defined molecular subtypes of bladder cancer (Lerner et al., 2016) to evaluate its potential usefulness as a biomarker for a defined subset of bladder cancer tissues.

Strikingly, we detected transcripts of OR10H1 not only in the bladder cancer tissues but also in the urine of bladder cancer patients, and again, there were significantly higher amounts in the urine of bladder cancer patients compared to the healthy test persons. There are no generally accepted tumor markers for bladder cancer. Even FDA-approved markers like nuclear matrix protein 22 (NMP22) (Soloway et al., 1996; Dal Moro et al., 2013; Chou et al., 2015; Yafi et al., 2015) are not sufficiently specific for routine clinical use. Based on our preliminary study, OR10H1 can certainly not yet be considered a tumor marker that is powerful enough to replace cystoscopy, but it might be used in combination with other RNA-biomarkers, in order to minimize false-positive diagnoses in patients presenting with hematuria, or to increase sensitivity during monitoring for recurrences.

Beside the identification of OR10H1 as a potential biomarker, we aimed to characterize its influence on bladder cancer cells. Since OR10H1 was an orphan receptor, we first concentrated on its deorphanization and identified Sandranol as an activating ligand. The natural occurring substances from sandalwood essential oils are used to mitigate irritable conditions of the bladder (Ballabh et al., 2008) and have been studied as chemopreventive agents for decades (Dwivedi and Abu-Ghazaleh, 1997). Santalols, the main constituents of sandalwood oil, inhibit and destabilize tubulin polymerization in oral cancer cells, prostate cells and breast cancer cells and cause apoptosis and cell cycle arrest in a concentration-dependent manner (Saraswati et al., 2013; Bommareddy et al., 2015; Lee et al., 2015). Therefore, we analyzed the effect of Sandranol on the cell cycle and cell morphology of BFTC905 cells and showed a reduction in adherens junction formation, as indicated by T-Cadherin staining. The immunocytochemical staining and microscopy further suggest that Sandranol stimulation affects the cytoskeleton further. As a response to the cytoskeletal changes, BFTC905 cells show a more rounded morphology upon Sandranol activation. This effect, like the stimulation of ATP and serotonin release, might also be relevant in the normal urothelium and needs to be analyzed in future studies using native tissues. Additionally, receptor activation by Sandranol in BFTC905 cells induced G1 arrest. In line with that, we observed a significant reduction in cell migration and proliferation and increased apoptosis, indicating the potential role of the receptor as a tumor target for bladder cancer. This is consistent with a study by Dozmorov, showing anti-proliferative and pro-apoptotic effects of sandalwood essential oils on human bladder cancer J82 cells and non-malignant UROtsa cells (Dozmorov et al., 2014). In that study, which did not consider OR10H1, no effects on the cytoskeleton were noted. However, sandalwood oils affected cell cycle checkpoint signaling and transcriptional regulation.

In our study, we identified cAMP- and calcium-dependent signaling in BFTC905 cells after stimulation by Sandranol. The canonical olfactory signaling is mediated by an increase of intracellular cAMP that leads to the opening of Ca^2+^- and Na^+^-conducting CNG channels in olfactory sensory neurons (Bakalyar and Reed, 1991). The following depolarization of the olfactory sensory neurons is enhanced by opening of Ca^2+^-activated Cl^-^-channels, leading to additional Cl^-^-efflux (Kleene and Gesteland, 1991; Rasche et al., 2010). This canonical olfactory signaling pathway is also activated by some ORs expressed in non-olfactory tissues (Busse et al., 2014; Massberg et al., 2015; Manteniotis et al., 2016). ORs are capable of coupling to different types of G proteins. The canonical signaling pathway comprises Gα_olf_, which -when activated- triggers the dissociation of the protein into an α and βγ subunit (Jones and Reed, 1989). A previous study by Scholz et al. (2016) demonstrated that another G protein alpha subunit (Gnao)-mediated pathway coexists with the canonical olfactory cAMP pathway. This pathway, demonstrated for Olfr73, can be triggered in a ligand-selective manner (Scholz et al., 2016). Further studies need to elucidate the exact signaling pathway involved in Sandranol activated BFTC905 cells.

Based on the fact that modulation of cytokines is important in cancer therapy, especially in the metaphylaxis of early stage bladder cancer (Kumari et al., 2017), we investigated the effect of OR10H1 activation on the synthesis of cytokines, which revealed a significant increase in IL10 and IL15 upon stimulation. Previous studies already demonstrated the effect of ORs on cytokines synthesis. OR1D2, an OR active in human airway smooth muscle cells, induces secretion of IL-8 upon stimulation by bourgeonal (Kalbe et al., 2016). The role of IL-10 in tumor biology is controversial; IL-10 is capable of suppressing the cellular immune response and might decrease tumor killing by the immune system (Halak et al., 1999), whereas other studies postulated an antitumor activity of IL10 in breast cancer (Kundu et al., 1996). Recent studies demonstrate the high potential of IL-15 for inducing natural killer cell expansion (van Audenaerde et al., 2017; Wagner et al., 2017) and decreasing blood vessel formation, cell migration and proliferation (Rohena-Rivera et al., 2017). The Sandranol induced increase in IL-15 might therefore contribute to the anti-proliferative and anti-migratory effects observed in our study, but could actually be more relevant for the effects of OR10H1 activation in normal and cancerous tissues *in vivo*.

In the present study, we describe a specific GPCR, OR10H1, belonging to the class of ORs, which is highly specific for the urinary bladder, but moreover has a significantly higher expression in bladder cancer tissues. Deorphanization and functional characterization of OR10H1 in BFTC905 cells revealed Sandranol as a specific agonist, which reduced proliferation, migration and cell viability and induced cell cycle arrest and some apoptosis. The Sandranol-induced signaling pathway is mediated by activation of an adenylyl cyclase and a concentration-dependent increase of cAMP. Furthermore, ligand stimulation led to the enhancement of ATP secretion and increased IL10 and IL15, which might be beneficial for the activation of natural killer cells *in vivo*. An important question for future research is how the immediate consequences of OR10H1 activation such as cAMP increase, calcium influx, ATP and serotonin secretion translate into the long-term consequences of cytoskeletal reorganization and inhibition of cell proliferation.

Our findings suggest that further studies should address the usefulness of OR10H1 as a potential tumor biomarker and target for therapy, to be used as a supportive approach in normal and cancerous urinary bladder tissues.

## Ethics Statement

The study was carried out in accordance with the Declaration of Helsinki. A written informed consent was signed by each patient who participated. All experiments were done in agreement with the ethics committee of the Faculty of Medicine, Ruhr University Bochum, Ethic number: 17-6116.

## Availability of Data and Materials

The datasets supporting the findings of this work are included within the article and its supplementary information.

## Author Contributions

LW, WS, HH, and GG: project conceptualization. LW, WS, SP, AT, GG, and HH: formal analysis. LW, WS, JE, BU, BK, SP, MH, and AT: investigation. LW, WS, and GG: manuscript writing (original draft preparation). JE, BU, MH, AT, HH, and SP: manuscript review and editing. LW, MH, SP, BK, AT, and JE: data visualization. LW, GG, AT, SP, and WS: supervision. LW and HH: overall project administration.

## Conflict of Interest Statement

The authors declare that the research was conducted in the absence of any commercial or financial relationships that could be construed as a potential conflict of interest.

## Supplementary Material

FIGURE S1 Immunocytochemical staining of OR10H1-transfected Hana3A cells. Staining of heterologously expressed OR10H1 in Hana3A cells using α-OR10H1 specific antibody (green, dilution: 1:50) and α-rho antibody (red, dilution 1:50). DAPI was used for visualization of the nuclei of Hana3A cells. Scale bar: 10 μm.

FIGURE S2 Amount of Serotonin (ng/ml) in the supernatant of Sandranol stimulated BFTC905 cells after 24 h. Cells were stimulated with Sandranol in the concentration 300, 500, and 700 μM.

FIGURE S3 Proliferation analyses by EdU labeling of BFTC905 cells after stimulation with Santalol (100 and 300 μM) for 48 h.

TABLE S1 Primers.

TABLE S2 Datasets analyzed.

## Abbreviations

DMSO: dimethylsulfoxid
OR: olfactory receptor
