# Supplementary figures and images for: Characterization of the Olfactory Receptor OR10H1 in Human Urinary Bladder Cancer
Source: Front Physiol. 2018 May 16;9:456. doi: 10.3389/fphys.2018.00456 (PMC5964926; doi:10.3389/fphys.2018.00456)

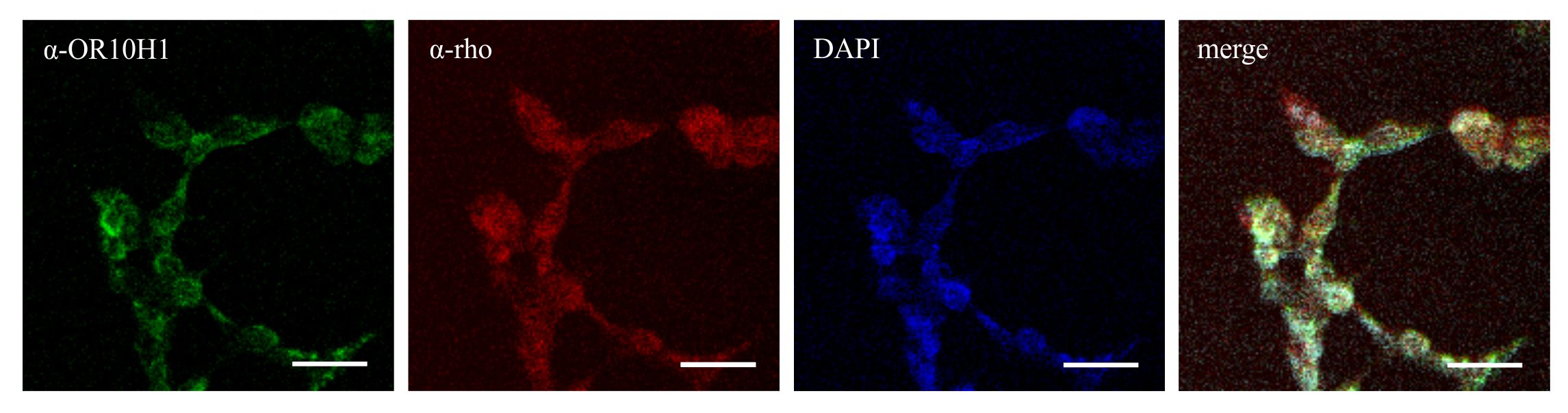

Supplement: FIGURE S1 — Immunocytochemical staining of OR10H1-transfected Hana3A cells. Staining of heterologously expressed OR10H1 in Hana3A cells using α-OR10H1 specific antibody (green, dilution: 1:50) and α-rho antibody (red, dilution 1:50). DAPI was used for visualization of the nuclei of Hana3A cells. Scale bar: 10 μm. [file Image_1.JPEG]

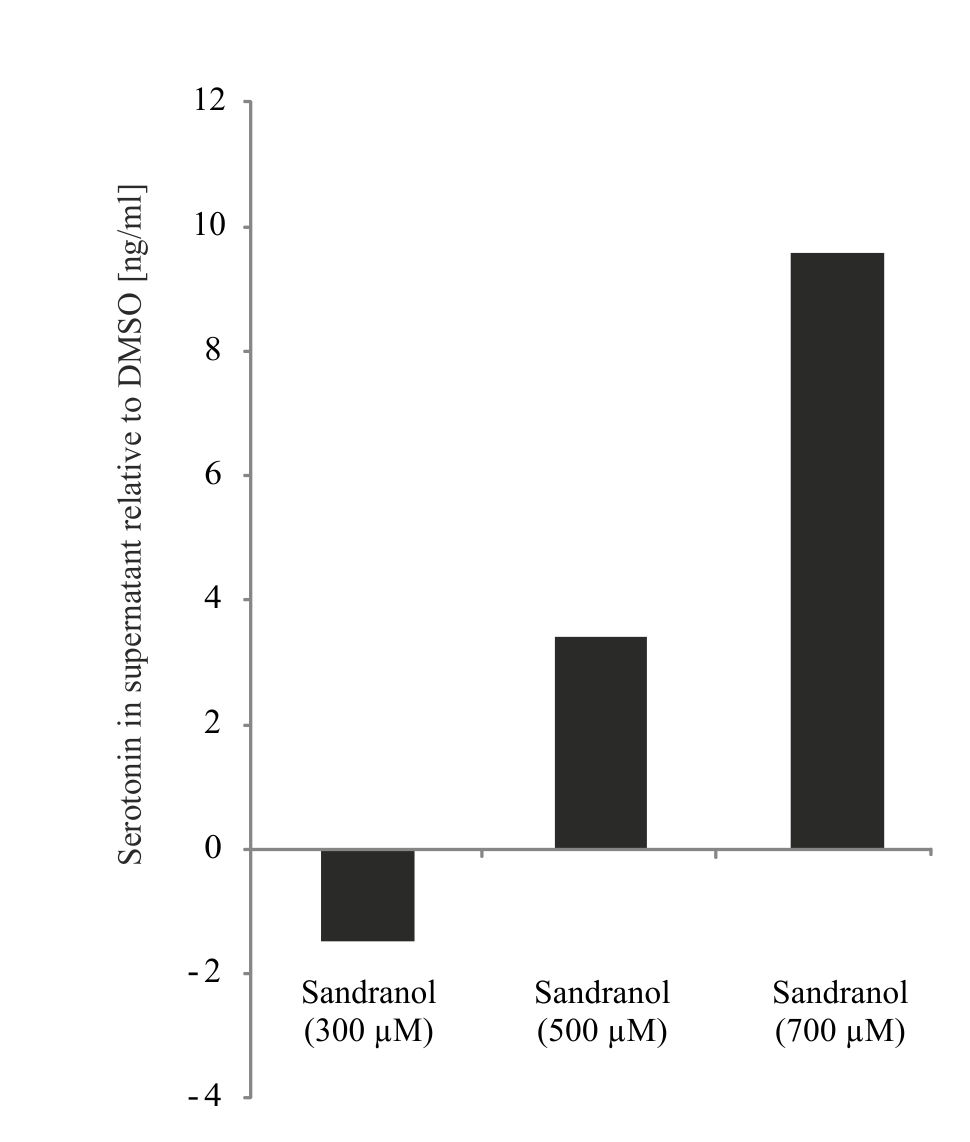

Supplement: FIGURE S2 — Amount of Serotonin (ng/ml) in the supernatant of Sandranol stimulated BFTC905 cells after 24 h. Cells were stimulated with Sandranol in the concentration 300, 500, and 700 μM. [file Image_2.JPEG]

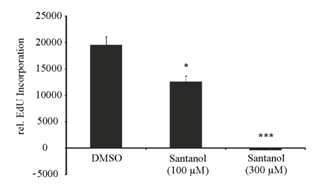

Supplement: FIGURE S3 — Proliferation analyses by EdU labeling of BFTC905 cells after stimulation with Santalol (100 and 300 μM) for 48 h. [file Image_3.JPEG]
